# Microfabrication of Microchannels for Fuel Cell Plates

**DOI:** 10.3390/s100100167

**Published:** 2009-12-28

**Authors:** Ho Su Jang, Dong Sam Park

**Affiliations:** Department of Mechanical Engineering, University of Incheon, Incheon 402-749, Korea; E-Mail: jang9714@inchen.ac.kr

**Keywords:** fuel cell separator, microchannel, microfabrication, powder blasting

## Abstract

Portable electronic devices such as notebook computers, PDAs, cellular phones, *etc.*, are being widely used, and they increasingly need cheap, efficient, and lightweight power sources. Fuel cells have been proposed as possible power sources to address issues that involve energy production and the environment. In particular, a small type of fuel-cell system is known to be suitable for portable electronic devices. The development of micro fuel cell systems can be achieved by the application of microchannel technology. In this study, the conventional method of chemical etching and the mechanical machining method of micro end milling were used for the microfabrication of microchannel for fuel cell separators. The two methods were compared in terms of their performance in the fabrication with regards to dimensional errors, flatness, straightness, and surface roughness. Following microchannel fabrication, the powder blasting technique is introduced to improve the coating performance of the catalyst on the surface of the microchannel. Experimental results show that end milling can remarkably increase the fabrication performance and that surface treatment by powder blasting can improve the performance of catalyst coating.

## 1. Introduction

Recently, portable electronic devices such as notebook computers, PDAs, cellular phones, camcorders, *etc.*, are being widely used, and they increasingly need cheap, efficient, and lightweight power sources. Fuel cells have been proposed as a possible power source to address issues that involve energy production and the environment. In particular, small fuel-cell systems are known to be suitable for portable electronic devices. The development of micro fuel cell systems can be achieved by the application of microchannel technology. Heat-transfer flow paths with a characteristic dimension in the range of 100–200 μm are classified as microchannels. The evolution of microchannel-based fuel cells or fuel processors has largely been triggered by advances in microfabrication technologies [1].

Many researchers are currently developing microchannel heat-exchangers, reactors, and separators as components for compact hydrogen generators for fuel cells. They are trying to develop low-cost materials and methods of fabrication for fuel cells. With the recent remarkable developments in high-technology industries that are based on information technology (IT), biotechnology (BT), nano technology (NT), *etc.*, micro-fabrication technology for ultra-micro parts with high precision and high functionality is required. It has been shown that this microfabrication technology can be effectively applied for the miniaturization of fuel cells. For the microfabrication of a small fuel-cell, MEMS technology that is based on conventional silicon-wafer technology has been used. Recently, chemical etching or mechanical micromachining that uses ultra-high-precision machine tools have been attempted for the fabrication of micro fuel cells [2,3].

In this study, the conventional method of chemical etching and the mechanical machining method of micro endmilling were used for the microfabrication of microchannels for a fuel cell separator, which is a key part of small fuel cells. The two methods were compared in terms of their performance in fabrication with regard to dimensional errors, channel flatness, channel straightness, and surface roughness of the channel. Further, the powder blasting technique, which is a sort of mechanical etching, was introduced to improve the coating efficiency of the catalyst on the surface of the microchannel that was fabricated by etching or micro endmilling.

## 2. Fabrication Methods of Microchannel

### 2.1. Chemical etching

Chemical etching has been the most general method for microchannel fabrication. This method needs a masking process for the selective removal of material. As this method is based on a chemical reaction, undercuts or overcuts can be generated depending on the density of the chemical material and the duration of etching. Therefore, etching cannot be used for the fabrication of a microchannel with precision.

### 2.2. Mechanical etching (powder blasting)

Powder blasting is a kind of mechanical etching process and can be applied with micro abrasives for machine micro shapes that are below 100 μm in size. In the process, micro abrasives are accelerated by highly compressed air or gases and collide with the workpiece with very high velocity and density. Thus, this process is capable of micromachining through the integration of brittle mode fractures that are based on the propagation of micro cracks. The removal of material can be accomplished by scanning the blasting nozzle along pre-defined paths on the workpiece that is covered with a mask. Since the shape of the machined workpiece can be determined by the mask pattern, very complex and/or micro shapes can be easily obtained [4–9]. Recently, CASIO Co. (Japan) developed a micro fuel processor of glass with microchannels using powder blasting [10]. Fuelcell Power Co. (Korea) has successfully fabricated microchannels on a bipolar plate [11]. It was shown that the microfabrication of channels through powder-blasting could enable small and light-weight bipolar plates.

### 2.3. Precision endmilling

With the development of high-precision machine technology, mechanical micro-machining has been studied for the fabrication of ultra-micro parts with high precision and high functionality [12]. It was shown that this microfabrication technology could be effectively applied to the miniaturization of fuel cells. For the microfabrication of a small fuel cell, MEMS technology based on the conventional silicon wafer technology have been used. Mechanical micromachining usually use CNC milling machine with ultra-precision position control. Precision endmilling can directly machine microchannels on the material(channel plate) without any preprocessing needed for chemical and mechanical etching processes.

## 3. Experimental Methods

### 3.1. Experimental samples

**Figure 1.**
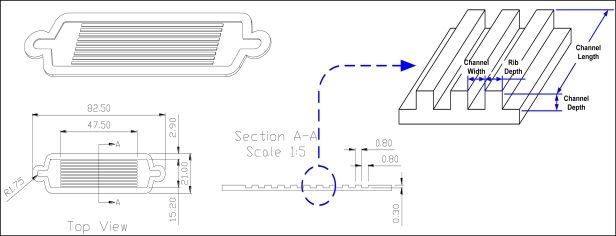
Shape and dimensions of the channel plate.

The material of the samples used for the channel-fabrication experiments was stainless steel 316 L. The shape and dimensions of the microchannel plate are shown in Figure 1 and Table 1. As a preliminary process for chemical etching and surface treating by powder-blasting, pattern design and pattern film masking processes were carried out. Through the masking process, channels, which were to be removed by chemical etching or endmilling, were determined. The optimum masking conditions were determined on the basis of the required experimental results.

### 3.2. Fabrication methods

For the microchannel fabrication experiments, five samples were prepared, as shown in Table 1. Cases 1, 2, 3, and 4 were fabricated through both chemical etching and endmilling, while Case 3-rl and 3-r2 were fabricated by only endmilling. Endmilling was performed through a CNC machining center. The depth of endmilling was setup to be up to 4–5 times the total channel depth to minimize machining-induced deformations due to the cutting forces.

### 3.3. Surface treating by powder blasting

**Figure 2.**
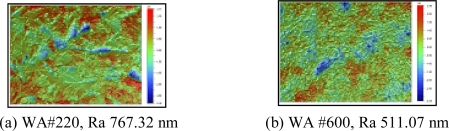
Surface roughness (0.15 MPa).

For the surface treatment of channels that are fabricated by chemical etching and endmilling, a microblaster (Sintoblaster, MB1 type) was used. Channels with masking film that was not removed were scanned by the blasting nozzle along the path, “ 
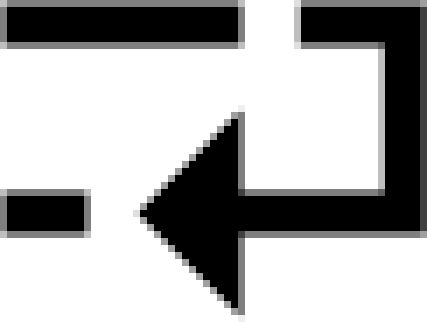
”. Before the main experiments, a series of preliminary experiments were performed to obtain the optimum blasting conditions by varying the required process parameters, as shown in Table 2. Based on the preliminary experiments, the most appropriate process parameters were decided. If the blasting pressure was higher than 0.2 MPa, experimental samples were twisted. The abrasive, WA#220, yielded a rougher surface than WA#600, as shown in Figure 2. Therefore, in the following, main experiments, WA#220 was used, and the blasting pressure was fixed at 0.15 MPa.

## 4. Experiments and Result Analysis

Fabrication experiments and analysis were carried out for all the samples shown in Table 1. In this paper, the experimental results for Case 4 are described. A contact-type form-detection device (FormTracer) and non-contact-type optical measuring devices (QV606PT and WYKO NT-1000) were used to measure and analyze the shape of the fabricated channel plate and the surface roughness of the channels.

### 4.1. Surface treating of etched channels

**Figure 3.**
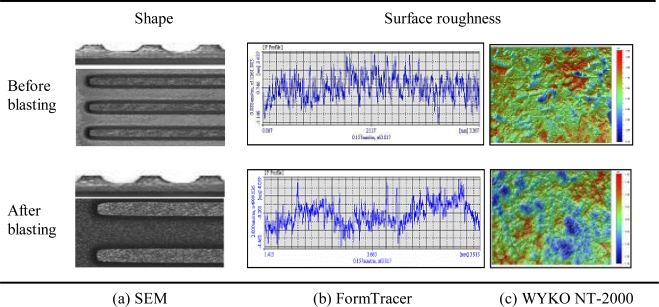
Shape and surface roughness of the etched sample.

Figure 3 shows the shape and surface roughness of the etched samples as well as those of the surface-treated samples. The shape of the channel profile was similar to the letter “U”. The experimental results are summarized in the 3^rd^ and 5^th^ columns of Table 3. The channel width, which was originally 0.8 mm in the mask pattern, increased by about 0.28 mm after etching. The maximum etching depth was about 0.337 mm.

The surface roughness of the etched channel was in the range of Ra 0.36 μm∼Ra 0.45 μm. For these etched samples, when surface treatment was carried out using powder-blasting, the channel depth, channel width, rib width, and channel straightness did not show remarkable changes after surface treatment. The channel flatness increased a little, which was thought to be due to the twisting deformation of the channel plate due to the blasting pressure. The surface roughness roughly doubled. This means the channel surface became uneven and rough, which would be a great help in increasing the coating efficiency of the catalyst.

### 4.2. Surface treating of channels fabricated by endmilling

**Figure 4.**
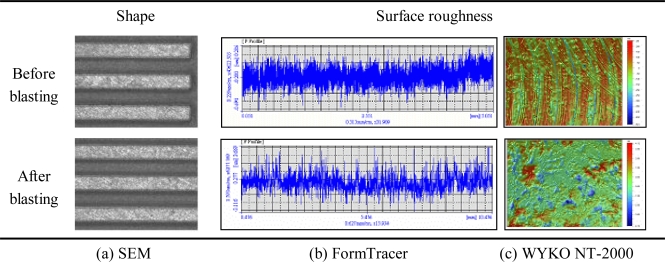
Shape and surface roughness of a sample that was fabricated by endmilling.

Figure 4 shows the shape and surface roughness of an etched channel and those of a surface-treated channel. The experimental results are summarized in the last two columns of Table 3. When channels were machined by endmilling, the dimensional errors of channels and ribs were about 0.02 mm each. The channel width, which was originally 0.8 mm in the mask pattern, increased by about 0.02∼0.0427 mm after endmilling.

The maximum milling depth was about 0.318 mm. The surface roughness of these channels was in the range of Ra 0.06 μm∼Ra 0.08 μm. The channel straightness was remarkably different in comparison with those of the etched samples. These experimental results imply that the endmilling method can remarkably improve dimensional precision, when compared with the chemical-etching method. Following surface-treatment, the channel flatness increased considerably, which is due to the large twisting deformation of the channel plate by the cutting force and the residual stress. The surface roughness was in the range of Ra 0.55∼Ra 0.78 μm. Through the powder-blasting of channels that were formed by endmilling, the surface roughness increased to about 10 times. This meant the channel surface became uneven and rough, which would be a great help in increasing the coating efficiency of the catalyst.

For endmilling and blasting of channels, fabrication time was about 5 minutes and 1 minute each, respectively. On the other hand, it was about 20 minutes for chemical etching. The cost of fabrication using endmilling and blasting is thought to be very low, considering time-consuming and high cost masking preprocess for chemical etching.

## 5. Summary

A conventional chemical-etching method and the endmilling method were used for microchannel fabrication for a small fuel cell separator. In addition, the powder-blasting technique for special surface treatment of the fabricated channel was introduced to increase the efficiency of the catalyst coating. The conclusions of this study could be summarized as follows:

1. The channel depth, which is a key factor in channel design, was almost similar for all the fabrication methods. In the case of etching, the width of the channel increased by about 0.28 mm, while the width of the rib decreased by about 0.3 mm. These large errors in fabrication imply that etching is not so good for channel fabrication.
2. When channels were machined by endmilling, the dimensional errors of channels and ribs were about 0.02 mm each. Endmilling could remarkably improve dimensional precision, when compared with chemical etching.
3. The channel straightness was shown to be similar in all the cases of the fabrication experiment. Although the channel flatness increased under endmilling, its absolute value was not so significant.
4. Through the powder blasting treatment on the channels that were formed by etching and endmilling, the surface roughness roughly doubled (in the case of etching) and increased up to 10 times (in the case of endmilling). This meant that the channel surface became uneven and rough, which would be a great help in increasing the coating efficiency of the catalyst.
5. From the results of this study, it could be concluded that the proposed method of endmilling, followed by powder blasting, could improve not only the dimensional precision but also the coating efficiency of the catalyst.

## Figures and Tables

**Table 1.** Dimensions of microchannel samples.

**Channel type**	Thickness*	Width*	Depth*	Rib width*	**No. of channels**
case 1	0.6	1.2	0.25	0.4	10
case 2	0.6	1	0.3	0.6	10
case3-r1	0.6	1	0.3	0.6	10
case3-r2	0.6	1.1	0.4	0.5	10
case 4	0.6	0.8	0.3	0.8	10

* in mm

**Table 2.** Conditions that were applied for powder blasting.

Sample material	316 L stainless steel
Abrasive	WA #220, WA #600
Mass flow rate (g/min)	60
Blasting pressure (MPa)	0.1, 0.15, 0.2, 0.25
Angle of impact (°)	90
Distance of nozzle (mm)	100
Nozzle diameter (mm)	8
Nozzle X speed (mm/s)	50
Nozzle Y speed (mm/s)	100
Nozzle pitch (mm)	5
Number of scans	1

**Table 3.** Comparison of several channel fabrication methods (CASE 4).

**Item(Case 4)**	**design spec.**	**etching**	**etching + blasting**	**endmilling**	**endmilling + blasting**
channel depth (mm)	0.3	0.337	0.338	0.318	0.340
channel width (mm)	0.8	1.073∼1.082	1.076∼1.083	*	0.815∼0.841
rib width (mm)	0.8	0.476∼0.495	0.485∼0.512	*	0.775∼0.786
Straightness (deviate.)	-	0.009∼0.200	0.010∼0.012	*	0.012∼0.014
Flatness (°)	-	−0.012	0.014	0.042	0.074
surface roughness (μm)	-	0.36∼0.45	0.7∼0.9	0.06∼0.08	0.55∼0.78

* Not measured as mask film was not removed for powder blasting
